# MiR-191 inhibit angiogenesis after acute ischemic stroke targeting VEZF1

**DOI:** 10.18632/aging.101948

**Published:** 2019-05-07

**Authors:** Kang Du, Can Zhao, Li Wang, Yue Wang, Kang-Zhen Zhang, Xi-Yu Shen, Hui-Xian Sun, Wei Gao, Xiang Lu

**Affiliations:** 1 Department of Geriatrics, Sir Run Run Hospital, Nanjing Medical University, Nanjing 211166, Jiangsu Province, China; 2 Key Laboratory for Aging and Disease, Nanjing Medical University, Nanjing 211166, Jiangsu Province, China; * Equal contribution

**Keywords:** acute stroke, angiogenesis factor, miR-191, VEZF1

## Abstract

Acute ischemic stroke (AIS) is a major public health problem in China. Impaired angiogenesis plays crucial roles in the development of ischemic cerebral injury. Recent studies have identified that microRNAs (miRNAs) are important regulators of angiogenesis, but little is known the exact effects of angiogenesis-associated miRNAs in AIS. In the present study, we detected the expression levels of angiogenesis-associated miRNAs in AIS patients, middle cerebral artery occlusion (MCAO) rats, and oxygen-glucose deprivation/reoxygenation (OGD/R) human umbilical vein endothelial cells (HUVECs). MiR-191 was increased in the plasma of AIS patients, OGD/R HUVECs, and the plasma and brain of MCAO rats. Over-expression of miR-191 promoted apoptosis, but reduced the proliferation, migration, tube-forming and spheroid sprouting activity in HUVECs OGD/R model. Mechanically, vascular endothelial zinc finger 1 (VEZF1) was identified as the direct target of miR-191, and could be regulated by miR-191 at post-translational level. In vivo studies applying miR-191 antagomir demonstrated that inhibition of miR-191 reduced infarction volume in MCAO rats. In conclusion, our data reveal a novel role of miR-191 in promoting ischemic brain injury through inhibiting angiogenesis via targeting VEZF1. Therefore, miR-191 may serve as a biomarker or a therapeutic target for AIS.

## INTRODUCTION

Acute ischemic stroke (AIS) is a major cerebrovascular disease ascribing to the sudden reduction of cerebral blood flow, characterized by a series of cellular and molecular disturbances. With a mortality rate of 10.3% and a morbidity rate of 19.7% in China [1], ischemic stroke is the leading cause of death and disability worldwide [2]. Several common risk factors, including hypertension, diabetes, dyslipidemia, alcohol, smoking, and inflammation [3–5], have been identified to be related to the pathogenesis of AIS. It is generally accepted that impaired neurovascular repair, especially angiogenesis, plays crucial roles in the development of ischemic cerebral injury [6].

Angiogenesis is the physiological process through which new blood vessels form by the extension or elaboration of existing vessels [7]. This process depending on endothelial cells is under the control of an extensive variety of angiogenic stimulators and inhibitors [8]. A growing number of studies have shown that microRNAs (miRNAs) are involved in the regulation of angiogenesis in ischemic diseases, including AIS [9–11].

MiRNAs are ~22nt RNAs that mediate posttranscriptional regulation of mRNA targets by binding to their 3′ un-translated region (UTRs) in diverse eukaryotic lineages [12]. Changes of MiRNA expression profile have been detected in AIS patients [13], middle cerebral artery occlusion (MCAO) rats [14] and oxygen-glucose deprivation/reoxygenation (OGD/R) cells [15–17]. Particularly, several angiogenesis-associated miRNAs, such as miR-210 [18, 19], miR-21 [20, 21], miR-126 [22, 23], miR-155 [24, 25], miR-31 [26], miR-223-3p [27], miR-191 [28] and miR-361 [29], are altered in patients with AIS and injured endothelial cells. However, the mechanism underlying the role of angiogenesis-associated miRNAs in AIS remains to be further explored.

Thus, the aim of the present study was to investigate the effects of angiogenesis-associated miRNAs in the angiogenesis after AIS both *in vitro* and *in vivo*.

## RESULTS

### Screening of miRNAs

To exclude tumor-related angiogenesis, we retrieved all published miRNAs related to the angiogenesis of endothelial cell in Pubmed (32 miRNAs, Table 1). After excluded 24 miRNAs that have been shown to play definite roles in acute stroke, we selected 8 miRNAs for further study (Table 2).

**Table 1:** Endothelial angiogenesis associated miRNAs.

**miRNA names**	**Cell types**	**miRNA target(s)**	**Impact on angiogenesis**	**References(PMID)**
miR-210	HUVEC	EFNA3	promote	18417479
miR-424	HUVEC;BOEC;MVEC	CUL2	promote	20972335
miR-200b	HMEC	ETS1	inhibit	21081489
miR-24	HUVEC	GATA2;PAK4	inhibit	21788589
miR-21	HPAEC	RhoB; Rho-kinase	promote	22371328
miR-125b	HUVEC	VE-cadherin	inhibit	22391569
miR-361	HUVEC	VEGF	inhibit	23128854
Let-7;miR-103	HUVEC	AGO1	promote	23426184
miR-29a	HUVEC	HBP1	promote	23541945
miR-93	HUVEC	P21; E2F-1; P53	promote	23559675
miR-320	HUVEC	NRP1	inhibit	24114198
miR-31	EPC	FAT4;TBXA2R	promote	24558106
miR-101	HUVEC	Cul3	promote	24844779
miR-221	EPC	PIK3R1	inhibit	25236949
miR-222	EPC	ETS1	inhibit	25236949
miR-223-3p	CMEC	RPS6KB1;HIF-1α	inhibit	25313822
miR-429	HUVEC	HIF-1α	inhibit	25550463
miR-107	RBMECs; HUVECs	Dicer-1	promote	26294080
miR-185	HMEC-1	STIM1	inhibit	26694763
miR-640	HUVEC	HIF-1α	inhibit	26879375
miR-140-5p	HUVEC	VEGF	inhibit	27035554
miR-126	EPC;ESC	SPRED1; PIK3R2/p85β	promote	27180261
miR-195	hEPC	GABARAPL1	inhibit	27623937
miR-155	HUVEC	E2F2	promote	27731397
miR-193a-3p	ECFC	HMGB1	inhibit	28276476
miR-186	HUVEC	HIF-1α	inhibit	28571741
miR-153-3p	HUVEC	HIF-1α	inhibit	28985553
miR-191	HUVEC	HIF-2α	inhibit	30090327
miR-503	EPC	Apelin	promote	29800588
miR-19b	HUVEC	TGFβ2	inhibit	30425199
miR-19a	HUVEC	EDNRB	inhibit	30550764

Abbreviations: HUVEC: human umbilical vein endothelial cells; BOEC: blood outgrowth endothelial cells; MVEC: microvasculature endothelial cells; HMEC: human microvascular endothelial cells; HPAECs: human pulmonary arterial endothelial cells; EPC: endothelial progenitor cells; CMEC: cardiac microvascular endothelial cells; RBMEC: rat brain microvascular endothelial cells; ESC: embryonic stem cell; ECFC: circulating endothelial colony forming cells; EFNA3: Ephrin-A3; CUL2/3: cullin 2/3; Ets-1: v-ets erythroblastosis virus E26 oncogene homolog 1; GATA2: Endothelial transcription factor GATA2; PAK4: Serine/threonine-protein kinase PAK4; VE- cadherin: vascular endothelial (VE)-cadherin; VEGF: vascular endothelial growth factor; AGO1: argonaute 1; HBP1:HMG-box transcription factor 1; P21: cyclin dependent kinase inhibitor 1A; E2F-1/2: E2F transcription factor 1/2; P53: tumor protein p53; NRP1: Neuropilin 1; FAT4: FAT atypical cadherin 4; TBXA2R: thromboxane A2 receptor; PIK3R1: phosphoinositide-3-kinase regulatory subunit 1; RPS6KB1: ribosomal protein S6 kinase B1; HIF-1/2α: hypoxia inducible factor 1/2 subunit alpha; STIM1: stromal interaction molecule 1; SPRED1: Sprouty-related protein; PIK3R2/p85β: phosphoinositol-3 kinase regulatory subunit 2; GABARAPL1: GABA type A receptor associated protein like 1; HMGB1: high mobility group box 1; TGFβ2: transforming growth factor beta 2; EDNRB: endothelin receptor type B.

**Table 2:** Endothelial angiogenesis associated miRNAs without AIS study.

**miRNA names**	**Cell types**	**miRNA target(s)**	**Impact on angiogenesis**	**References**
miR-361	HUVEC	VEGF	inhibit	[25]
miR-29a	HUVEC	HBP1	promote	[26]
miR-31	EPC	FAT4;TBXA2R	promote	[22]
mir-223-3p	CMEC	RPS6KB1;HIF-1α	inhibit	[23]
miR-640	HUVEC	HIF-1α	inhibit	[27]
miR-193a-3p	ECFC	HMGB1	inhibit	[28]
miR-191	HUVEC	HIF-2α	inhibit	[24]
miR-503	EPC	Apelin	promote	[29]

Abbreviations: AIS: acute ischemic stroke; HUVEC: human umbilical vein endothelial cells; EPC: endothelial progenitor cells; CMEC: cardiac microvascular endothelial cells; ECFC: circulating endothelial colony forming cells; VEGF: vascular endothelial growth factor; HBP1:HMG-box transcription factor 1; FAT4: FAT atypical cadherin 4; TBXA2R: thromboxane A2 receptor; RPS6KB1: ribosomal protein S6 kinase B1; HIF-1/2α: hypoxia inducible factor 1/2 subunit alpha; HMGB1: high mobility group box 1.

### Characteristics of enrollment patients

We enrolled 6 AIS patients and 6 control subjects as Cohort A, and another 12 AIS patients and 12 control subjects as Cohort B. Characteristics of Cohort A and B were shown in Table 3 and Table 4, respectively. There was no statistically difference of demographic or vascular risk factors between AIS patients and controls.

**Table 3:** Characteristics of acute ischemic stroke and control subjects in Cohort A.

**Characteristic**	**Healthy controls (M±SEM)**	**Stroke patients (M±SEM)**	***P* value**
Age (years)	74.5±3.9	71.3±4.2	0.592
Gender (%)	50F/50M	50F/50M	1
CAD history, n(%)	2(33.3)	1(16.7)	1
HT history, n(%)	5(83.3)	3(50)	0.545
DM history, n(%)	2(33.3)	2(33.3)	1
Smoking history, n(%)	1(16.7)	1(16.7)	1
BMI(kg/m^2^)	26.8±1.3	25.2±0.6	0.297
SBP(mmHg)	145.7±8.6	148.3±7.5	0.820
DBP(mmHg)	73.5±5.8	74.2±3.6	0.204
mRS score	2.67±0.8	-	-
HBA1c(%)	6.5±0.5	6.8±0.5	0.694
FBG(mmol/L)	5.7±0.5	6.6±0.6	0.307
TG(mmol/L)	1.3±0.3	1.6±0.6	0.719
TC(mmol/L)	4.4±0.7	4.7±0.5	0.789
LDL-C(mmol/L)	2.5±0.5	2.8±0.4	0.617
HDL-C(mmol/L)	1.6±0.3	1.4±0.2	0.732
Creatinine(μmol/L)	80.3±9.0	73.8±7.1	0.584
BUN(mmol/L)	5.6±0.4	6.2±0.8	0.526
LP(α)(mg/L)	249.3±120.1	288.3±139.0	0.836
Hcy(μmol/L)	15.8±2.1	13.8±0.8	0.341
AST(U/L)	18.4±2.3	17.9±1.5	0.861
ALT(U/L)	21.4±3.0	16.6±2.6	0.256
γGT(U/L)	41.8±14.5	16.8±1.7	0.118
CRP(mg/L)	2.4±1.1	4.7±3.0	0.481
WBC count(×10^9^/L)	5.7±0.7	6.2±0.6	0.605
Hemoglobin(g/L)	133.5±6.3	139.3±5.7	0.507
Platelets(×10^9^/L)	169.7±13.3	177.8±32.5	0.821
PT(s)	11.5±0.6	10.5±0.5	0.230
APTT(s)	33.1±2.0	30.1±2.0	0.311
FIB(g/L)	2.9±0.2	3.0±0.1	0.609

Abbreviations: CAD: coronary artery disease; HT: hypertension; DM: diabetes mellitus; BMI: body mass index; SBP: systolic blood pressure; DBP: diastolic blood pressure; HBA1c: glycolated hemoglobin A1c; FBG: fasting blood glucose; TG: total triglyceride; TC: total cholesterol; LDL-C: low density lipoprotein cholesterol; HDL-C: high density lipoprotein cholesterol; BUN: blood urea nitrogen; LP(α): lipoprotein; Hcy: homocysteine; AST: aspartate aminotransferase; ALT: alanine aminotransferase; γGT: γ-aminobutyric acid; CRP: C-reactive protein; WBC: white blood cell; PT: prothrombin time; APTT: activated partial thromboplastin time; FIB: fibrinogen.

**Table 4:** Characteristics of acute ischemic stroke and control subjects in Cohort B.

**Characteristic**	**Healthy controls (M±SEM)**	**Stroke patients (M±SEM)**	***P* value**
Age (years)	75.4±2.8	75.3±3.0	0.968
Gender (%)	50F/50M	48.7F/51.3M	1
CAD history, n(%)	2(16.7)	2(16.7)	1
HT history, n(%)	8(66.7)	9(75)	1
DM history, n(%)	2(16.7)	3(25)	1
Smoking history, n(%)	3(25)	4(33.3)	1
BMI(kg/m^2^)	24.7±0.7	23.2±1.2	0.273
SBP(mmHg)	131.8±5.0	135.1±4.2	0.614
DBP(mmHg)	72.5±3.4	76.7±2.9	0.357
mRS	2.58±0.8	-	-
HBA1c(%)	5.8±0.2	5.9±0.8	0.838
FBG(mmol/L)	6.0±0.6	7.5±1.1	0.246
TG(mmol/L)	1.4±0.1	1.7±0.3	0.205
TC(mmol/L)	4.2±0.2	4.6±0.2	0.212
LDL-C(mmol/L)	2.7±0.2	2.9±0.2	0.411
HDL-C(mmol/L)	1.2±0.1	1.3±0.1	0.578
Creatinine(μmol/L)	82.8±8.8	88.8±8.6	0.631
BUN(mmol/L)	6.7±1.3	6.8±1.0	0.926
LP(α)(mg/L)	181.4±54.8	273.6±137.3	0.545
Hcy(μmol/L)	18.9±3.2	17.2±2.6	0.741
AST(U/L)	23.8±2.7	24.5±2.8	0.860
ALT(U/L)	20.3±2.6	20.0±2.8	0.925
γGT(U/L)	53.1±23.7	43.4±10.3	0.713
CRP(mg/L)	15.0±9.2	19.8±12.3	0.759
WBC count(×10^9^/L)	7.0±0.9	6.9±1.1	0.890
Hemoglobin(g/L)	125.2±5.7	116.6±4.3	0.242
Platelets(×10^9^/L)	177.3±17.2	262.9±59.0	0.177
PT(s)	12.5±1.0	11.4±0.2	0.317
APTT(s)	33.7±1.5	31.9±1.1	0.355
FIB(g/L)	3.2±0.2	4.0±0.6	0.256

Abbreviations: CAD: coronary artery disease; HT: hypertension; DM: diabetes mellitus; BMI: body mass index; SBP: systolic blood pressure; DBP: diastolic blood pressure; HBA1c: glycolated hemoglobin A1c; FBG: fasting blood glucose; TG: total triglyceride; TC: total cholesterol; LDL-C: low density lipoprotein cholesterol; HDL-C: high density lipoprotein cholesterol; BUN: blood urea nitrogen; LP(α): lipoprotein; Hcy: homocysteine; AST: aspartate aminotransferase; ALT: alanine aminotransferase; γGT: γ-aminobutyric acid; CRP: C-reactive protein; WBC: white blood cell; PT: prothrombin time; APTT: activated partial thromboplastin time; FIB: fibrinogen.

### Expressions of miRNAs in AIS patients

**Figure 1:**
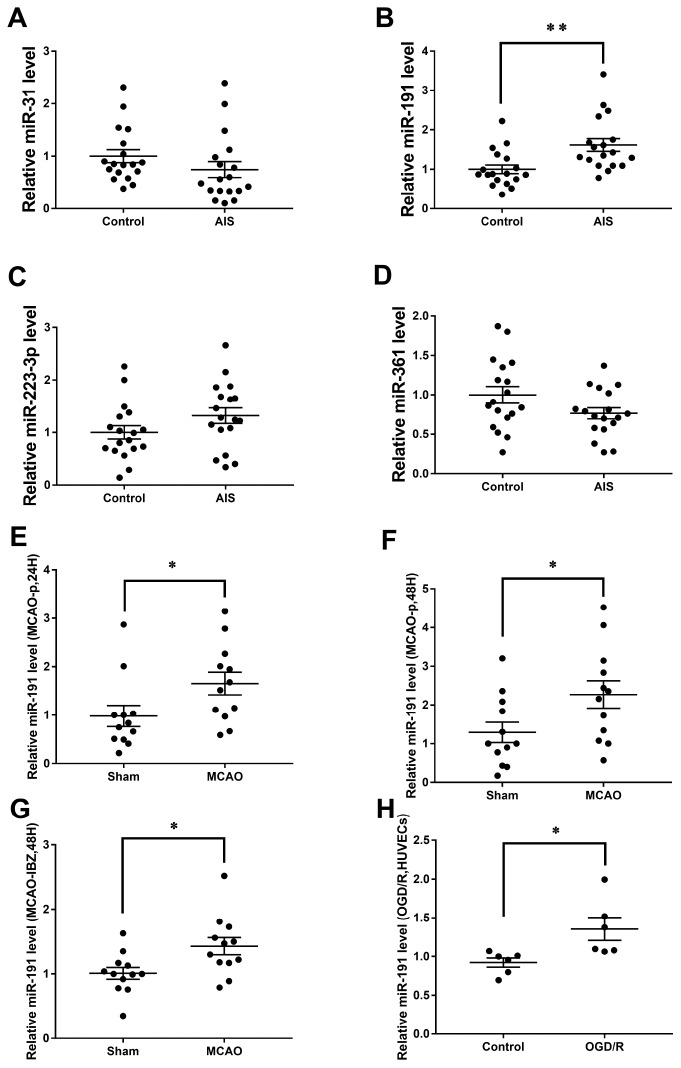
**Relative miRNAs levels.** Expression levels of miRNAs in Cohort A+B (n=18) (**A**) miR-31, (**B**) miR-191, (**C**) miR-223-3p, (**D**) miR-361; (**E**) Expression level of miR-191 in rat MCAO plasma after 24h reperfusion (n=12); (**F**) Expression level of miR-191 in rat MCAO plasma after 48h reperfusion (n=12); (**G**) Expression level of miR-191 in rat MCAO brains (n=12); (**H**) Expression level of miR-191 in OGD HUVECs (n=6). Means ± SEM. * P< 0.05,** P< 0.01 vs. NCm or NCi.

**Chart 1:**
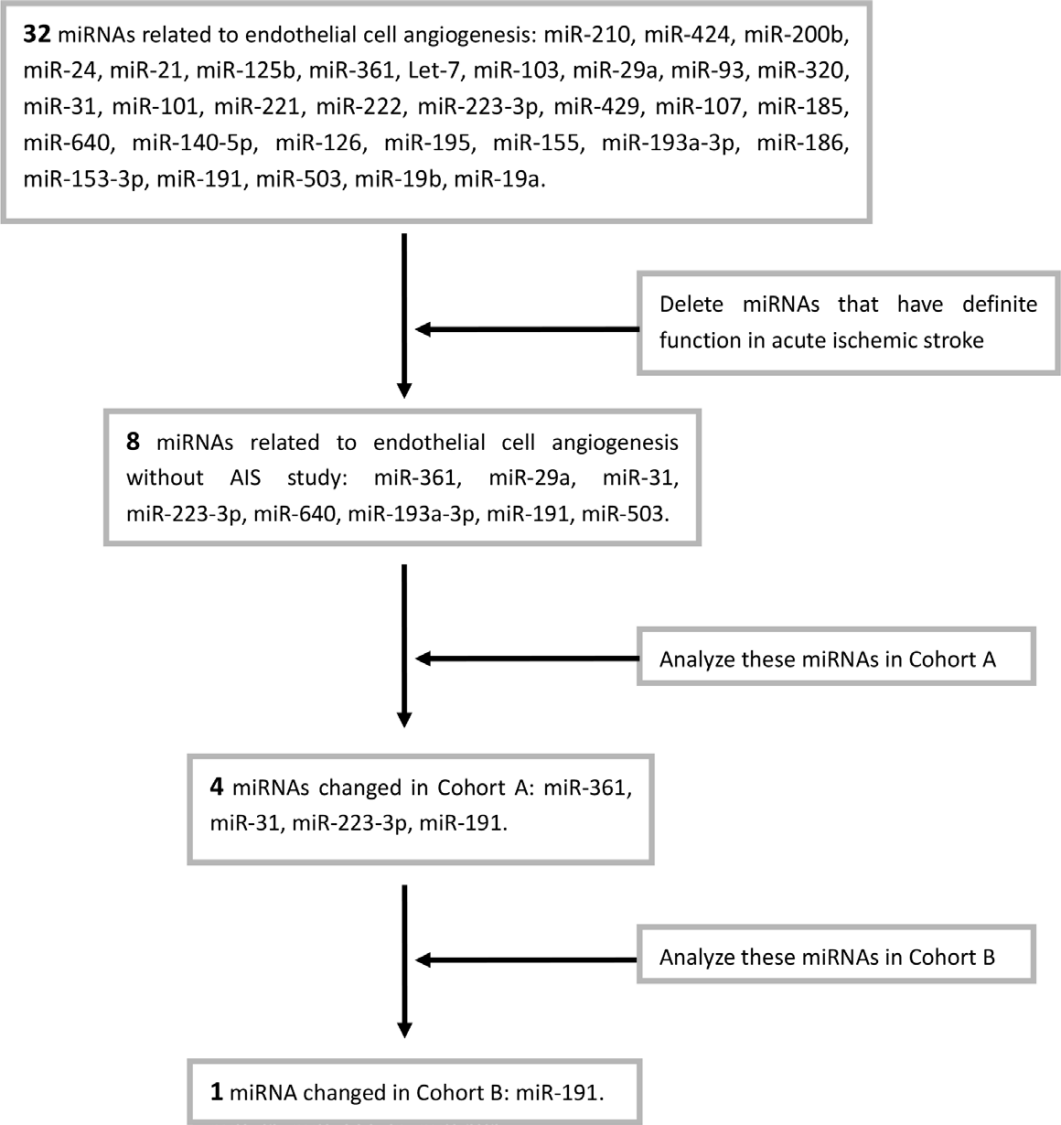
Screening process of miRNAs.

Eight angiogenesis-associated miRNAs (miR-361 [29], miR-29a [30], miR-31 [26], miR-223-3p [27], miR-640 [31], miR-193a-3p [32], miR-191 [28], miR-503 [33]) levels were first measured in Cohort A. The expression of miR-361, miR-31, miR-223-3p, and miR-191 were changed in AIS patients when compared to the controls (Supplementary Figure 1B, 1C, 1E, 1F). These four miRNAs, including miR-31 (Figure 1A), miR-191 (Figure 1B), miR-223-3p (Figure 1C), and miR-361 (Figure 1D) were further detected in Cohort B. . However, only miR-191 was higher in AIS patients than those controls (Figure 1B). The Chart 1 showed the screening process.

### Expression of miR-191 in rat MCAO model and OGD/R HUVECs

Consistently, miR-191 levels were increased in the plasma of rat MCAO model both at 24h and 48h after reperfusion [14, 34] (Figure 1E, 1F). However, no significant difference of miR-191 levels was observed between the two time points (data not shown). The expression of miR-191 was also increased in the ischemic boundary zone (IBZ) (Figure 1G). We further detected miR-191 in HUVECs and found that miR-191 expression was elevated in OGD/R group (Figure 1H).

### Function of miR-191 in HUVECs proliferation

**Figure 2:**
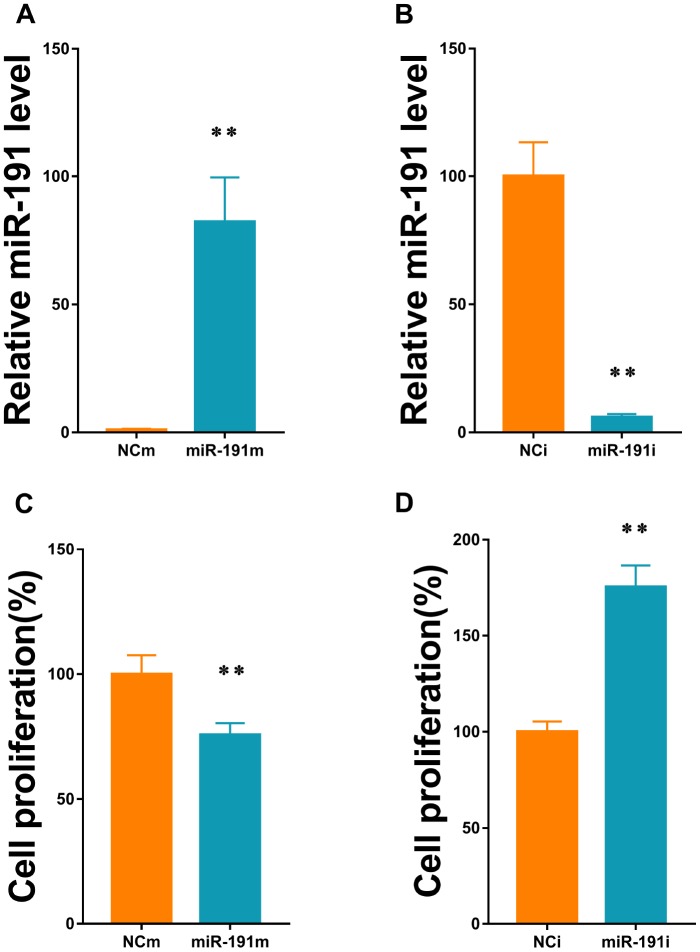
**MiR-191 transfection efficiency and cell proliferation.** (**A**, **B**) Expression level of miR-191 (in fold of NCm or percentage of NCi) in HUVECs that were transfected with miR-191 mimic (**A**) or miR-191 inhibitor (**B**) as assessed by real-time PCR (n=6 per group); (**C**, **D**) Proliferation (percentage of NCm or NCi) of HUVECs transfected with miR-191 mimic (**C**) or miR-191 inhibitor (**D**) as assessed by CCK-8 assay (n =6 per group). After transfection, the cells were reseeded into 96-well plates and incubated for another 48 h. Means ± SEM. ** P< 0.01 vs. NCm or NCi.

We transfected HUVECs with 50nM miR-191 mimic and 100nM miR-191 inhibitor to up and down-regulate the expression of miR-191, respectively (Figure 2A, 2B). HUVECs were then subjected to reoxygenation for 18h after 2h of OGD. We found that up-regulation of miR-191 significantly reduced HUVEC proliferation (Figure 2C), while down-regulation of miR-191 promoted the proliferation (Figure 2D).

### Function of miR-191 in HUVECs apoptosis and cell cycle

**Figure 3:**
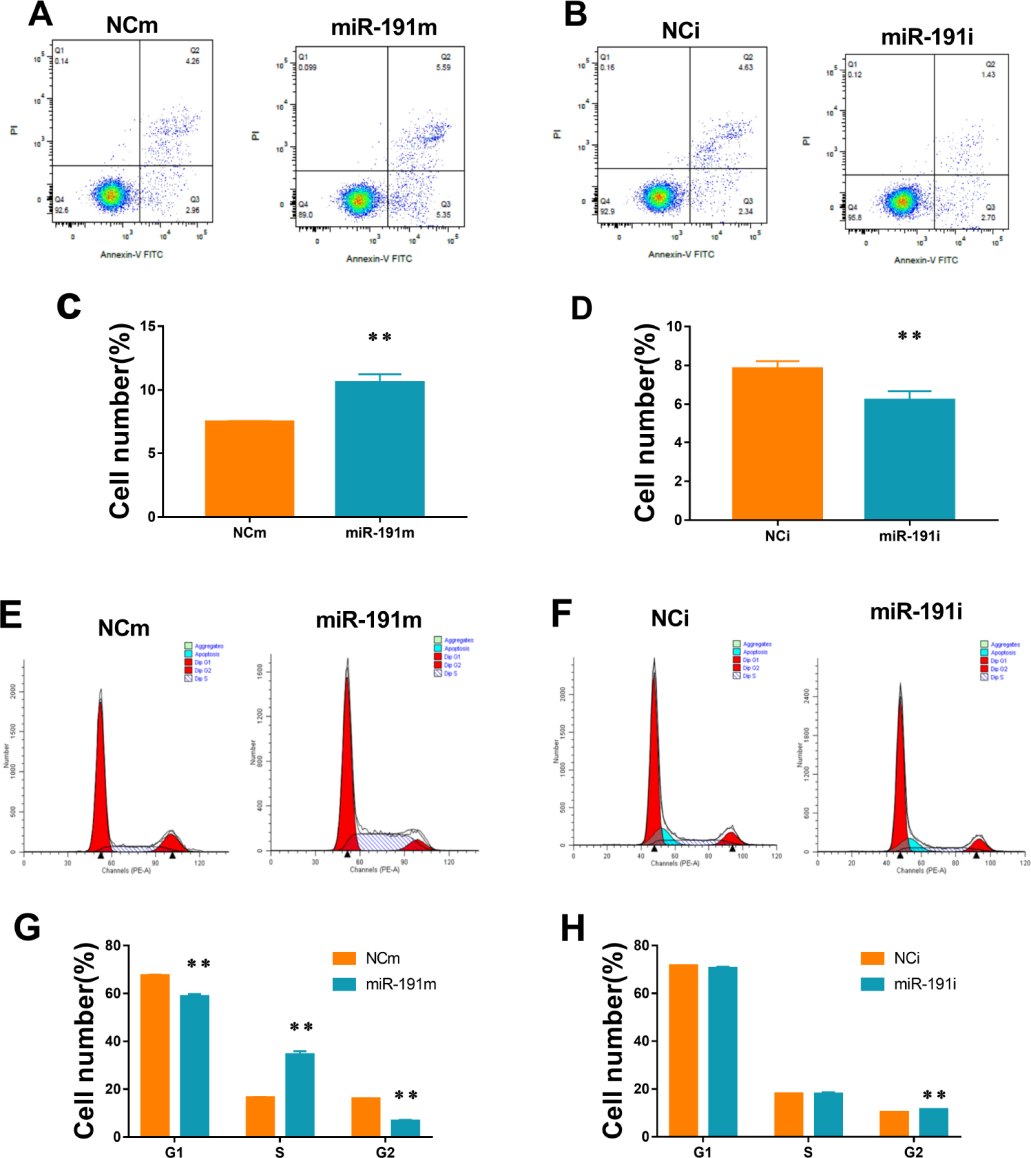
**Cell apoptosis and cell cycle.** (**A**, **B**) Cell apoptosis analysis of HUVECs transfected with miR-191 mimic (**A**) or miR-191 inhibitor (**B**)as assessed by flow cytometry; (**C**, **D**) Percentage of apoptosis HUVECs transfected with miR-191 mimic (**C**) or miR-191 inhibitor (**D**) (n =6 per group); (**E**, **F**) Cell cycle analysis of HUVECs transfected with miR-191 mimic (**E**) or miR-191 inhibitor (**F**) as assessed by flow cytometry; (**G**, **H**) Percentage of HUVECs transfected with miR-191 mimic (**G**) or miR-191 inhibitor (**H**) in different stages (n=6 per group). Means ± SEM.,** P< 0.01 vs. NCm or NCi.

By using flow cytometry, we showed that up-regulation of miR-191 increased the apoptosis rate of HUVECs (Figure 3A, 3C), while down-regulation of miR-191 ameliorated the apoptosis induced by OGD/R (Figure 3B, 3D). We also found that over-expression of miR-191 blocked the cell cycle in the S phase (Figure 3E, 3G), which is consistent with the study of Gu, Y., et al. [35]. However, silence of miR-191 only slightly increased the number of G2 cells (Figure 3F, 3H).

### Function of miR-191 in HUVECs migration

**Figure 4:**
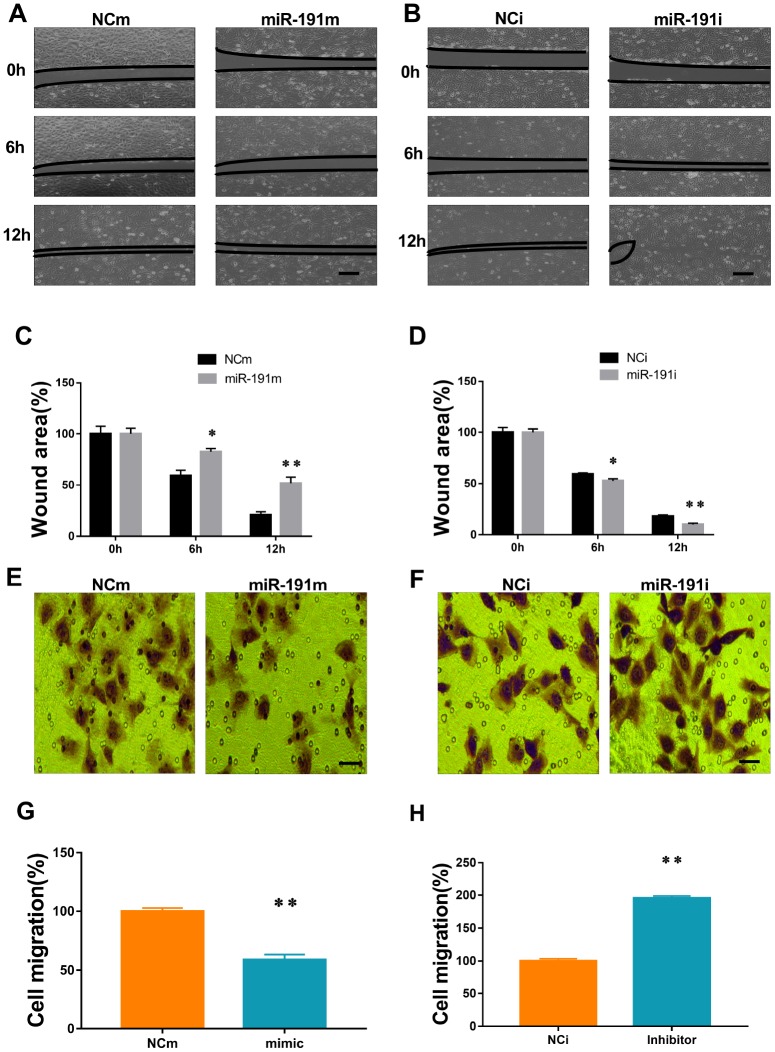
**MiR-191 inhibited cell migration.** (**A**, **B**) Phase contrast microscopic images of HUVECs at 0, 6, and 12 h after scratching. The cells were transfected with miR-191 mimic (**A**), miR-191 inhibitor (**B**), or the corresponding scrambled NCm (**A**) and NCi (**B**). Black lines indicate the wound area. Scale bars, 100 μm. (**C**, **D**) Size of wound area (percentage of 0 h) created by scratching HUVECs transfected with miR-191 mimic (**C**) or miR-191 inhibitor (**D**) (n =6 per group). (**E**, **F**) Phase contrast microscopic images of HUVECs migrated and attached to the bottom membrane of a transwell. The cells were transfected with miR-191 mimic (**E**), miR-191 inhibitor (**F**), or the corresponding scrambled NCm (**E**) and NCi (**F**). Scale bars, 20μm. (**G**, **H**) Number of migrated HUVECs (percentage of NCm or NCi) transfected with miR-191 mimic (**G**) or miR-191 inhibitor (**H**) (n =6 per group). Means ± SEM. * P< 0.05,** P< 0.01 vs. NCm or NCi.

To investigate the function of miR-191 in HUVECs migration in OGD/R, scratch wound healing assay and transwell migration assay were performed. Over-expression of miR-191 significantly delayed the closure of scratch wounds (Figure 4A, 4C) and markedly reduced the number of migrated cells (Figure 4E, 4G). In contrast, inhibition of miR-191 promoted the healing of scratch wounds (Figure 4B, 4D) and enhanced cell migration (Figure 4F, 4H).

### Function of miR-191 in HUVECs tube-forming activity

**Figure 5:**
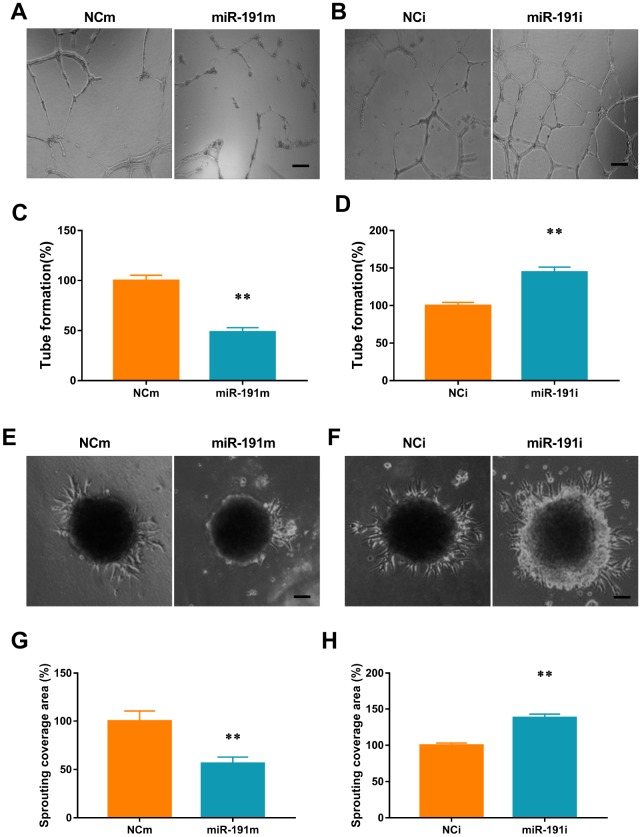
**miR-191 inhibited tube formation and spheroid sprouting. (A**, **B**) Phase-contrast microscopic images of tubeforming HUVECs that were transfected with miR-191 mimic (**A**), miR-191 inhibitor (**B**), or the corresponding scrambled NCm (**A**) and NCi (**B**). Scale bars, 20μm. (**C**, **D**) Tube formation of HUVECs (percentage of NCm or NCi) transfected with miR-191 mimic (**C**) or miR-191 inhibitor (**D**) as assessed by tube formation assay (n =6 per group). (**E**, **F**) Phase-contrast microscopic images of sprouting HUVECs spheroids. HUVECs were transfected with miR-191 mimic (**E**), miR-191 inhibitor (**F**), or the corresponding scrambled NCm (**E**) and NCi (**F**). Scale bars, 20μm. (**G**, **H**) Sprouting coverage area of HUVECs (percentage of NCm or NCi) transfected with miR-191 mimic (G) or miR-191 inhibitor (H) as assessed by spheroid sprouting assay (n = 6 per group). Means ± SEM. ** P< 0.01 vs. NCm or NCi.

We found that transfection of HUVECs with miR-191 mimic reduced the number of newly developed tube meshes when compared with controls (Figure 5A, 5C). In contrast, transfection of cells with miR-191 inhibitor promoted tube formation (Figure 5B, 5D)

### Function of miR-191 in HUVECs spheroid sprouting activity

We performed a 3-dimensional spheroid sprouting assay and demonstrated that over-expression of miR-191 significantly decreased the sprouting coverage area of HUVECs spheroids when compared with controls (Figure 5E, 5G). In contrast, transfection of cells with miR191 inhibitor markedly enhanced the sprouting coverage area (Figure 5F, 5H).

### Validation of predictive target gene of miR-191

**Figure 6:**
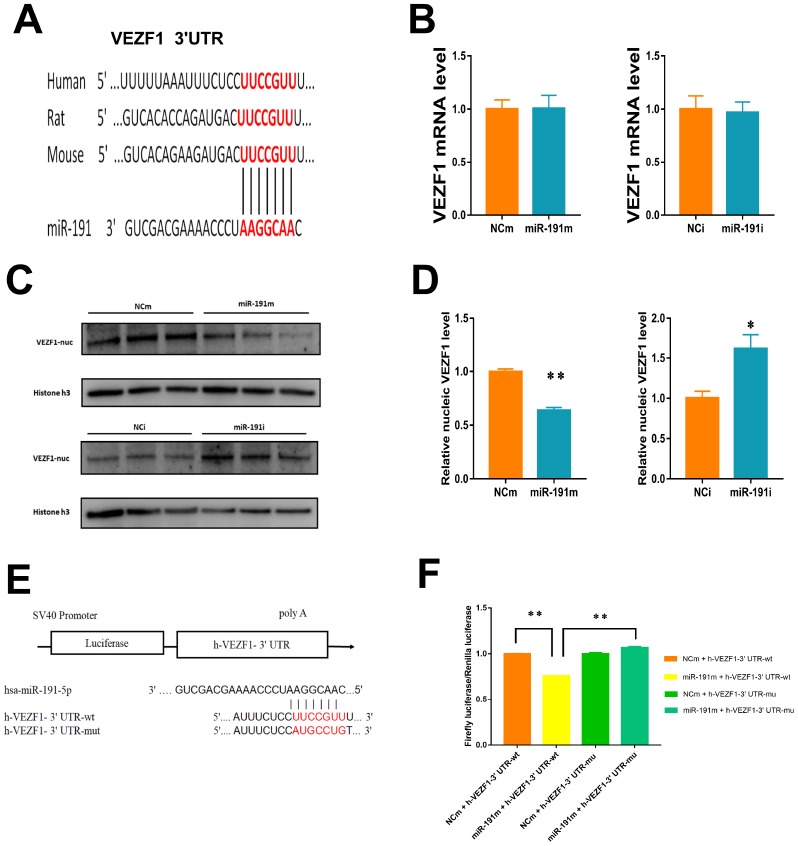
**Regulation of VEZF1 by miR-191.** (**A**) MiR-191 and its putative binding sequence in the 3'-UTR of VEZF1. (**B**) Real-time PCR analysis of VEZF1 expression in HUVECs by miR-191 interference. (n =6 per group); (**C**, **D**) Western blot analysis of VEZF1 expression in HUVECs transfected by miR-191. (**E**) Construction of Plasmid; (**F**) Luciferase activities were measured to evaluate the binding of miR-191 to the candidate binding sequence of VEZF1 after transfection of miR-191 mimic or NCm. Means ± SEM. * P< 0.05,** P< 0.01 vs. NCm or NCi.

Vascular endothelial zinc finger 1 (VEZF1) is one of the target genes of miR-191 predicted by TargetScan 7.2 and miRDB (Figure 6A). We found that VEZF1 mRNA levels were not influenced by miR-191 mimic or miR-191 interference (Figure 6B). Since VEZF1 is a nucleus transcription factor, we then extracted nucleoprotein and found that over-expression of miR-191 decreased VEZF1 protein levels in nucleus, while miR-191 inhibitor increased the protein levels of VEZF1 (Figure 6C, 6D). Further luciferase assay showed a significant decrease in the luciferase activity of wild-type VEZF1 3’ UTR (Figure 6E, 6F), indicating that VEZF1 is the target of miR-191.

### Action of miR-191 on VEZF1 signaling

**Figure 7:**
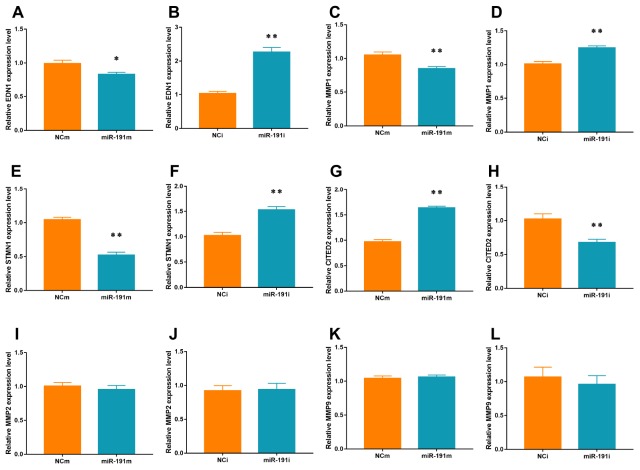
**mRNA levels of Targets of VEZF1.** Real-time PCR for EDN1 (**A**, **B**), MMP1 (**C**, **D**), STMN1 (**E**, **F**), CITED2 (**G**, **H**), MMP2 (**I**, **J**), and MMP9 (**K**, **L**) mRNA of HUVECs transfected with miR-191 mimic or miR-191 inhibitor (n = 6 per group). Means ± SEM. * P< 0.05,** P< 0.01 vs. NCm or NCi.

VEZF1 has been reported to regulate several genes that are involved in angiogenesis, such as endothelin 1 (EDN1) [36], matrix metalloproteinase 2 (MMP2) [36], stathmin 1 (STMN1) [37], matrix metalloproteinase (MMPs) [38], Cbp/p300 interacting transactivator with Glu/Asp rich carboxy-terminal domain 2 (CITED2) [39]. In the present study, we found that miR-191 over-expression significantly suppressed the mRNA levels of EDN1, MMP1, and STMN1 and but increased the mRNA level of CITED2 (Figure 7A, 7C, 7E, 7G). Inhibition of miR-191 showed an opposite trend of the changes of these genes (Figure 7B, 7D, 7F, 7H). We did not find any effects of miR-191 on the mRNA levels of MMP2 and MMP9 (Figure 7I–7L).

### Inhibition of miR-191 reduced infarction volume of MCAO rats

**Figure 8:**
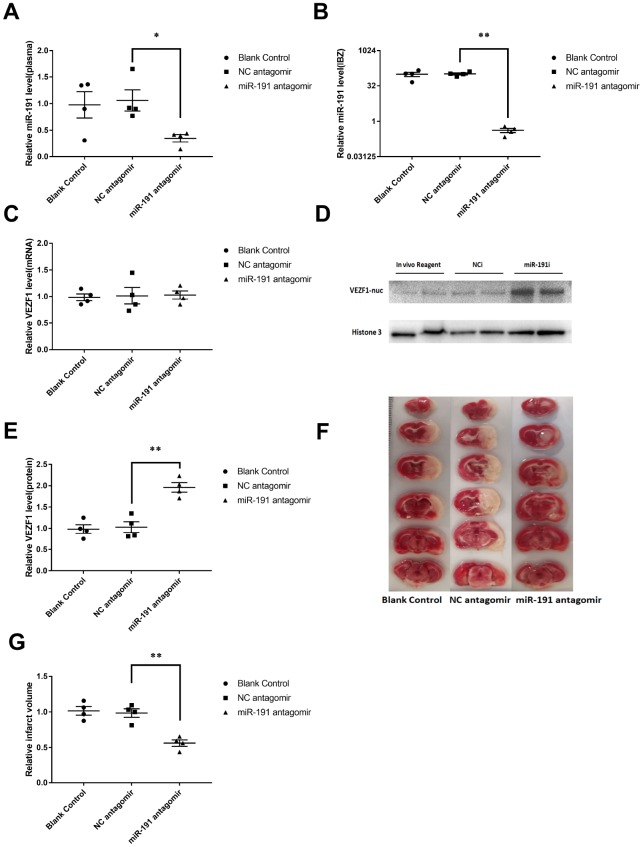
**Inhibition of miR-191 reduced infarction volume of MCAO rats after the injection of miR-191 antagomir compared to NC antagomir and blank control.** Relative miR-191 levels in (**A**) plasma and (**B**) IBZ (n =4 per group); (**C**) Real-time PCR analysis of VEZF1 expression in IBZ (n =4 per group); (**D**, **E**) Western blot analysis of VEZF1 expression in IBZ (n =4 per group); (**F**, **G**) Relative infarct volume of MCAO rats (n =4 per group). Means ± SEM. * P< 0.05,** P< 0.01 vs. NC antagomir.

The rats randomly received an intracerebroventricular infusion of miR-191/NC antagomir or blank control 3 days prior to MCAO. Compared to the NC antagomir, miR-191 antagomir significantly reduced the miR-191 levels both in plasma and IBZ at 48 h after reperfusion in MCAO rats (Figure 8A, 8B). We found that VEZF1 mRNA levels of IBZ were not influenced by miR-191 antagomir (Figure 8C) which was consistent with the results of cell experiments. However, the protein levels of VEZF1 were increased significantly (Figure 8D, 8E). We also found that rats receiving miR-191 antagomir had smaller brain infarct volumes than those with NC antagomir (Figure 8F, 8G).

## DISCUSSION

Impaired angiogenesis plays a crucial role in cerebral injury after acute ischemic attack [6]. MiRNAs have been shown to be important regulators involved in the process of angiogenesis [10, 40]. Here we showed for the first time that miR-191 was elevated in the plasma of AIS patients as well as in MCAO rat model and OGD/R HUVEC model. Over-expression of miR-191 promoted apoptosis but inhibited proliferation, migration, tube-forming and spheroid sprouting activity in HUVECs, while silence of miR-191 displayed opposite results. Mechanistically, we found that miR-191 directly regulated VEZF1, leading to the changes of a variety of angiogenesis-associated genes targeted by VEZF1. *In vivo* studies demonstrated that Inhibition of miR-191 could reduce the infarction volume induced by MCAO in rats. Therefore, miR-191 might be a novel therapeutic target for the treatment of AIS.

Angiogenesis is one of the key repair mechanisms for the ischemic injury induced by acute stroke [7]. Gu *et al.* [35] demonstrated that miR-191 was preferentially expressed in endothelial cells compared to other types of human cells and displayed antiangiogenic effect. In the present study, we performed a series of well-established angiogenesis assays and demonstrated that miR-191 is an inhibitor of angiogenesis with effects of suppressing proliferation, migration, tube formation and spheroid sprouting in HUVECs. Knockdown of miR-191 could reduce the infarction area induced by MCAO in rats and promote proliferation, migration, tube formation and spheroid sprouting in HUVEC. Our results complement nicely with a previous report showing that knockout of miR-191 reduced hepatic ischemia-reperfusion injury through inhibiting inflammatory responses and cell death [41]. Another study also showed that up-regulated miR-191 participated in renal ischemia-reperfusion injury via inducing apoptosis of renal tubular epithelial cells [42]. These results indicate that lowing miR-191 might be a potential therapy for ischemia-reperfusion injury. However, although we found that plasma levels of miR-191 were increased in patients with AIS, there might be false positive and negative results in the process of miRNA screening because of the relative small sample size. Future studies with larger sample size will be needed to verify the exact roles of miR-191 in the diagnosis and prediction of AIS.

VEZF1 encodes a zinc finger transcription factor which is essential for developmental angiogenesis and lymphangiogenesis. Mammalian VEZF1 is expressed in the anterior-most mesoderm at E7.5 during development and is later restricted in the vascular endothelium [43]. VEZF1 knockout mice showed embryonic lethality caused by vascular remodeling defects and loss of vascular integrity, indicating that VEZF1 is a critical regulator of vascular development [44]. VEZF1 is thus proposed to act as a transcriptional activator of pro-angiogeneic genes including EDN1 [36], MMP2 [36], STMN1 [37], MMPs [38], CITED2 [39]. VEZF1 can be epigenetically regulated by histone acetylation and deacetylation [43]. VEZF1 is specifically expressed in endothelial cells and correlated with the differentiation and proliferation of endothelial cells in the embryonic vascular system [45]. A series of studies showed that VEZF1 could activate angiogenesis by promoting endothelial cells proliferation, migration and vessel network formation [37, 39, 46, 47]. Our data demonstrated that VEZF1 is regulated by miR-191 at post-translational level. Luciferase reporter assay validated that VEZF1 is a direct target of miR-191. To further prove miR-191 inhibit HUVECs angiogenesis by targeting VEZF1, we measured the expression levels of the VEZF1 targets [36–39] after miR-191 interference. Our data shown that over-expression of miR-191 inhibited the expression of angiogenesis-related genes including EDN1, MMP1, and STMN1. However, further intervention studies are needed to elucidate whether miR-191 inhibited angiogenesis via targeting VEZF1.

**Figure 9:**
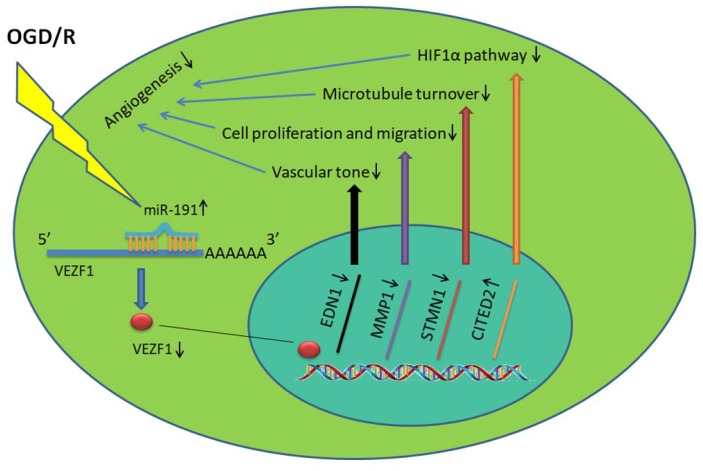
**miR-191 reduces the nucleoprotein VEZF1, resulting in up-regulation of CITED2 and down-regulation of MMP-1, STMN1 mRNA.** This, in turn, suppresses HUVEC proliferation and migration and thus prevents endothelial tube formation and spheroid sprouting. Moreover, miR-191 suppresses the expression of EDN1 which functions as maintaining vascular tension.

In conclusion, our data reveal a novel role of miR-191 in promoting ischemic brain injury through inhibiting angiogenesis via targeting VEZF1, which in turn resulted in up-regulation of CITED2 and down-regulation of MMP-1, STMN1.(Figure 9). MiR-191 might be served as a promising effective biomarker and therapeutic target for AIS.

## METHODS

### Ethics statement

Investigation was conducted in accordance with the ethical standards and according to the Declaration of Helsinki and was approved by the Ethics Committee of Sir Run Run Hospital, Nanjing Medical University (Protocol Numbers: 2018-SR-25, Supplementary Figure 2) and the Animal Ethical and Welfare Committee of Nanjing Medical University (Protocol Numbers: IACUC-1806010, Supplementary Figure 3).

### Patient enrollment

Patients were recruited consecutively from the department of geriatrics of Sir Run Run Hospital, Nanjing Medical University from January to June 2018. There were total 18 patients with AIS and 18 controls. AIS patients were recruited from stroke center within six hours from the onset of the symptoms before thrombolytic therapy. AIS diagnosis was confirmed using clinical features and brain MRI by two investigators in a double-blinded manner according to the 2018 Guidelines for the Early Management of Patients With Acute Ischemic Stroke [2]. The control subjects were selected during the same period in the same hospital from the health examination center. Exclusion criteria including: under 18 or over 90 years old; history of intracranial hemorrhage; craniocerebral trauma; giant intracranial aneurysms; recent (within 3 months) history of intracranial surgery; malignant tumors. Informed consents were obtained from all subjects.

### MCAO

Male Sprague Dawley rats (7-8w, 230 g–280 g) randomly divided into two groups (12 in each group) were obtained from Shanghai Sippr-BK laboratory animal Co. Ltd. Right MCAO was induced using an intraluminal filament as Longa, E.Z. *et al* [48] described. The body temperature was maintained at 37 °C with a homothermal blanket and physiological parameters were monitored during the surgical procedure. After anesthetized with 1 % pentobarbital sodium (0.5ml/100g), the right common carotid artery (CCA), external carotid artery (ECA) and internal carotid artery (ICA) were sequentially isolated. An incision was made in the distal region of the CCA, then a 40mm long (diameter: 0.26-0.28mm) poly-L-lysine coated nylon monofilament (Beijing Shadong Biotechnology Co., Ltd.) was inserted into the ICA, and the monofilament was advanced approximately 18-20mm beyond the carotid bifurcation until mild resistance was encountered. The occlusion was sustained for 2 h. The sham group underwent similar procedures, but the monofilament was not advanced into the CCA.

Neurological evaluations were performed according to an established graded scoring system at 24 h after reperfusion to verify the modeling success in function [49]. Briefly, neurological deficits were scored as follows: 0, no deficit; 1, failure to extend left forepaw upon lifting the whole animal by tail; 2, grip strength weakening of the left forepaw; 3, circling to the left when held by the tail; and 4, spontaneous circling.

Peripheral venous blood was collected at 24 and 48 hours after reperfusion and the brains were removed and frozen at −20 °C for 15 min at 48 hours after reperfusion when sacrificed (Supplementary Figure 3). To histologically verify the success of the model, coronal sections were cut into 2 mm thick slices, stained with 1% 2,3,5-triphenyltetrazolium chloride (TTC) at 37 °C in the dark for 20 min, and photographed (Supplementary Figure 4).

### Plasma and brain fragments collection and storage

Peripheral blood samples were collected in tubes anticoagulated with ethylenediamine tetraacetic acid (EDTA) dipotassium salt after a definite diagnosis before any medication is administered. The samples were centrifuged (1000×g, 5minutes, 4 °C, Beckman Coulter) to remove blood cells and debris, and then transferred to 1.5ml microtubes (Axygen, MCT-150-C) for storage at –80°C until further processing. Brain fragments were collected and stored in liquid nitrogen.

### Primary HUVECs isolation and culture

Fresh umbilical cords were obtained following delivery of healthy babies to healthy mothers. HUVECs were isolated from umbilical cords according to a previously described method [50]. Briefly, the umbilical vein was inserted an intravenous needle for single use with tube and flushed with 30ml 37 °C phosphate buffer saline (PBS), following which the cord was clamped at the distal end and the vessel filled with collagenase (Type Ia, 1 mg/mL, Sigma, C9891), until mildly distended. Following incubation at 37°C for 10-15 min, the cord was unclamped and the digest drained. The vessel was gently massaged and flushed through with 20ml endothelial cell medium (ECM, Science, 1001), and the digests were pooled. The endothelial cell suspension was centrifuged (500×g, 5 min), and the cell pellet was resuspended in ECM. This suspension was seeded into a 25ml flask (Corning, 430639).

The cells were incubated at 37°C, 5% CO_2_ until they are 85% - 90% confluent. Then, cells were detached from the substratum by exposure to 0.25% trypsin-EDTA (Gibco, 25200072, US) for 1 min at 37°C, pelleted (500×g, 5 min) and passaged at a ratio of 1:3-5. Confluent cells at passage 3-6 were used for all experiments.

### OGD/R

The OGD/R protocol was performed to mimic ischemia *in vitro*. Briefly, culture medium was removed and rinsed with PBS for three times. HUVECs were placed into a tri-gas incubator (memmert, Eastern Friesland, Germany) containing 1% O_2,_ 5% CO_2_, 94% N_2_ at 37 °C with glucose-free Dulbecco's Modified Eagle Medium (DMEM, Gibco, 11966025, US). After two hours challenge, DMEM was replaced with ECM. The cells were maintained for further 24 h at 37 °C in a humidified 5% CO_2_ incubator to generate reperfusion.

### Total RNA isolation

Total RNA was isolated from plasma, brain and cell samples using TRIzon (CWBio, CW0580) reagent following the manufacturer’s instructions. RNA concentration and purity were determined with one drop spectrophotometer (OD-1000+, Nanjing wuyi Science and Technology Co., Ltd., China).

### Quantitative real-time polymerase chain reaction (qRT-PCR)

CDNA was generated from 1 μg RNA using miRNA 1^st^ Strand cDNA Synthesis Kit (by stem-loop) (vazyme, MR101-02, Nanjing, China) for miRNA or PrimeScript™ RT reagent Kit (Perfect Real Time) (Takara, RR047A, Japan) for mRNA. Real-time PCR was performed using QuantStudio 5 (Applied Biosystems, US) with miRNA Universal SYBR qPCR Master Mix (vazyme, MQ101-02) for miRNA or Maxima SYBR Green/ROX qPCR Master Mix (2X) (Thermo Scientific™, K0221, US) for mRNA according to the manufacturer’s protocol. All reactions were run in triplicate and relative gene expression was calculated using the comparative threshold cycle (Ct) method (relative gene expression = 2− ^(ΔCtsample-ΔCtcontrol)^). The U6 snRNA was used as an internal control for miRNA while TATA-binding protein (TBP) used for mRNA. The gene-specific primers sequences are listed in Table 5.

**Table 5:** Primer sets for real-time PCR analyses.

**Gene**	**Forward primer (5′ to 3′)**	**Reverse Primer (5′ to 3′)**
U6 (MIM:180692)	CTCGCTTCGGCAGCACA	AACGCTTCACGAATTTGCGT
TBP (NM_003194)	CCACTCACAGACTCTCACAAC	CTGCGGTACAATCCCAGAACT
VEZF1 (NM_007146)	GGACAGCTATCACCTGAGGC	GCGATGGTAGAGATAAGGGGAA
MMP1 (NM_002421)	AAAATTACACGCCAGATTTGCC	GGTGTGACATTACTCCAGAGTTG
MMP2 (NM_004530)	TACAGGATCATTGGCTACACACC	GGTCACATCGCTCCAGACT
MMP9 (NM_004994)	TGTACCGCTATGGTTACACTCG	GGCAGGGACAGTTGCTTCT
EDN1 (NM_001168319)	AGAGTGTGTCTACTTCTGCCA	CTTCCAAGTCCATACGGAACAA
CITED2 (NM_006079)	CCTAATGGGCGAGCACATACA	GGGGTAGGGGTGATGGTTGA
STMN1 (NM_203401)	TCAGCCCTCGGTCAAAAGAAT	TTCTCGTGCTCTCGTTTCTCA

**miRNA**	**Accession**	**Reverse transcription primers (5′ to 3′)**	**Forward primers (5′ to 3′)**
miR-29a	MIMAT0004503	GTCGTATCCAGTGCAGGGTCCGAGGTATTCGCACTGGATACGACCTGAAC	GCGCGACTGATTTCTTTTGGT
miR-31	MIMAT0000089	GTCGTATCCAGTGCAGGGTCCGAGGTATTCGCACTGGATACGACAGCTAT	GCGAGGCAAGATGCTGGC
miR-138	MIMAT0000430	GTCGTATCCAGTGCAGGGTCCGAGGTATTCGCACTGGATACGACCGGCCT	GCGAGCTGGTGTTGTGAATC
miR-191	MIMAT0000440	GTCGTATCCAGTGCAGGGTCCGAGGTATTCGCACTGGATACGACCAGCTG	CGCAACGGAATCCCAAAAG
miR-193a-3p	MIMAT0000459	GTCGTATCCAGTGCAGGGTCCGAGGTATTCGCACTGGATACGACACTGGG	CGCGAACTGGCCTACAAAGT
mir-223-3p	MIMAT0000280	GTCGTATCCAGTGCAGGGTCCGAGGTATTCGCACTGGATACGACTGGGGT	GCGCGTGTCAGTTTGTCAAAT
miR-361	MIMAT0000703	GTCGTATCCAGTGCAGGGTCCGAGGTATTCGCACTGGATACGACGTACCC	GCGCGTTATCAGAATCTCCAG
miR-503	MIMAT0002874	GTCGTATCCAGTGCAGGGTCCGAGGTATTCGCACTGGATACGACCTGCAG	CGTAGCAGCGGGAACAGTT
miR-640	MIMAT0003310	GTCGTATCCAGTGCAGGGTCCGAGGTATTCGCACTGGATACGACAGAGGC	CGCGATGATCCAGGAACCT

Abbreviation: TBP: TATA-box binding protein; MMP1/2/9: matrix metallopeptidase 1/2/9; EDN1: endothelin 1; CITED2: Cbp/p300 interacting transactivator with Glu/Asp rich carboxy-terminal domain 2; STMN1: stathmin 1.

### Transfection of miR-191 mimic or inhibitor into HUVECs

The cells were transfected for 24 h with 50 nM miR-191 mimic (Ribobio, Guangzhou, China) or 100 nM miR-191 inhibitor (Ribobio) using Lipofectamine 3000(Invitrogen, US) according to the manufacturer’s protocol. Cells transfected with negative control of mimic (NCm) (Ribobio) or negative control of inhibitor (NCi) (Ribobio) served as controls.

### Cell proliferation assay

To assess the proliferation rate of HUVECs, Cell Counting Kit-8(CCK-8) assays (Dojindo, Japan) were performed according to the manufacturer’s instructions. Briefly, 3000 HUVECs in 100 μl of cell suspension were seeded in 96-well flat-bottomed plates. After 8 hours incubation, CCK-8 reagent was added to each well, and the absorbance of each well was measured at 450 nm after 3 hours incubation by a microplate reader (Synergy H1, BioTek, US). A value of 100% was assigned to the respective control group.

### Flow cytometric analysis of apoptosis

HUVECs were treated with miR-191mimic/ihibitor for 24 h, and then subjected to OGD/R for 2 h/18 h. The apoptotic cell death rate was examined with Annexin V-FITC and PI double staining using the Annexin V-FITC apoptosis detection kit (Beyotime Biotechnology, C1063, Shanghai, China) according to the manufacturer’s instructions. Briefly, 5×10^4^ cells were collected with 0.25% EDTA free trypsin (Gibco, 15050065, US), washed and resuspended in PBS. After staining with Annexin V-FITC/PI, flow cytometric analysis was performed and data were analyzed using FlowJo software.

### Flow cytometric analysis of cell cycle

HUVECs were treated with miR-191 mimic/inhibitor for 24 h, and then subjected to OGD/R for 2 h/18 h. HUVECs were collected and fixed in 70% ethanol overnight at 4 °C by using the cell cycle detection kit (KeyGen BioTech, KGA512, Jiangsu, China). Single-cell suspensions were labeled with PI for 30 min at 4 °C and analyzed by flow cytometry (BD, New York, US). The data were analyzed with FlowJo software.

### Cell migration assay

Scratch wound healing assay and transwell migration assay were performed to evaluate the motility of HUVECs. For the scratch wound healing assay, 5×10^5^ of HUVECs were seeded in a 6-well plate. After reaching confluence, the cell monolayer was scratched with a pipette tip (10 μl) to generate 4 scratch wounds and then rinsed twice with PBS to remove nonadherent cells. Phase-contrast light micrographs were taken immediately after scratching (0 h) as well as after 6h and 12 h with ×200 magnification using a CKX41 microscope (Olympus, Japan).

For the transwell migration assay, 2×10^5^ of HUVECs in 500μl 1% FBS ECM were seeded into a 24-well insert (costar, 3422), and 750μl of 15% FBS ECM was added to the lowerwell. After 24h of incubation, nonmigrated cells were removed with cotton swabs, and migrated cells were stained with 0.1% crystal violet (Solarbio, C8470, Beijing, China). The number of migrated cells was determined in 3 microscopic regions of interest at ×200 magnification using a CKX41 microscope.

### Tube formation assay

To analyze the function of miR-191 in the tube-forming activity of HUVECs, 50 μl of Matrigel (8-12mg/ml; Corning) was plated in each well of a 96-well plate. After Matrigel polymerization, 3×10^4^ of HUVECs in 100μl ECM were added into each well. After 12 h, vessel-like network structures were examined under CKX41 microscope. Tube formation was quantified by measuring the number of meshes using ImageJ software [National Institutes of Health (NIH), Bethesda, MD, USA].

### Spheroid sprouting assay

HUVECs were suspended in ECM containing 0.25% (w/v) methylcellulose (Sigma-Aldrich) and seeded (1000 cells/100μl) in low attachment, round-bottom, 96-well spheroid microplates (Corning, 4520). After incubation for 24 h, spheroids were harvested and resuspended in 20μl Matrigel. The spheroid-containing Matrigel was rapidly transferred to 24-well plates and allowed to polymerize for 30 min, after which 500μl ECM was added to each well. After 24h of incubation, the spheroid-sprouting capacity was quantified by measuring the sprouting coverage area of the sprouts using imageJ software.

### Luciferase reporter assays

293T cells (3 × 10^5^ cells per well) were plated onto 24-well plates. pSV40-VEZF1-wt or pSV40-VEZF1-mut was cotransfected with miRNA-191 mimic or NCm into 293T cells by Lipofectamine 3000. The relative luciferase activity was normalized to Renilla luciferase activity 48h after transfection.

### Protein extraction and Western blots

Nuclear extracts were prepared with NE-PER™ Nuclear and Cytoplasmic Extraction Reagents according to the manufacturer's protocol (Thermo Scientific, 78833, US). Protein concentrations were measured by BCA protein assay (Beyotime Biotechnology, Shanghai, China). Equal amounts (20 μg) of protein were separated by 4-20% GenScript SurePAGE, Bis-Tris, precast polyacrylamide gels (GenScript Biotechnology, Nanjing, China) electrophoresis and transferred to polyvinylidene fluoride membranes (Millipore, Billerica, MA, USA). Membranes were then incubated overnight at 4 °C with a 1:1000 dilution of anti- Histone H3 (Cell Signaling Technology, 4499s, US) and anti-VEZF1 (Proteintech, 19003-1-AP, US). After additional incubation with a 1:5000 dilution of anti-rabbit IgG (heavy and light chain) antibody (CST, 7074S) for 2 h, the immune complexes were detected by Immobilon Western HRP Substrate Peroxide Solution (Millipore Corporation, Billerica, MA 01821, USA). And images were acquired using ChemiDoc^TM^ XRS+ Imaging System (Bio-rad, US). The intensity of immunoreactivity was assessed using Image Lab 6.0 software.

### Intracerebroventricular injection of the miR-191 antagomir

The miR-191 antagomir and NC antagomir were purchased from RiboBio(Guangzhou, China). The NC and miR-191 antagomir (2.5 μg/2.5 μl) were diluted with 1.25 μl of Entranster^TM^ in vivo transfection reagent (Engreen,18668-11-1, Beijing, China). The solution was mixed with 1.25μl PBS gently, kept at room temperature for 5 min and then injected intracerebroventricularly (i.c.v.) using a microsyringe (KD Scientific Inc., USA) under the guidance of a stereotaxic instrument (RWD Life Science). A solution of 3.75μl PBS added with 1.25 μl of Entranster^TM^ in vivo transfection reagent was acted as blank control. Intracerebroventricular injection was performed according to a previously described method [51]. The stereotaxic coordinates the right lateral ventricle: ML: -1.40mm, AP: -0.36mm, DV: -3.90mm.

### Statistics

Differences between the two groups were analyzed by the unpaired Student’s *t* test or Mann-Whitney test after testing the distribution of the data. Differences between multiple groups were analyzed by one-ANOVA followed by the Student-Newman-Keuls *post hoc* test (Graphpad Prism 7.0, USA) after testing the data for equal variance. All values were expressed as mean ± SEM. Statistical significance was accepted at P<0.05.

## Supplementary Material

Supplementary Figures
