# Supplementary material for: MiR-191 inhibit angiogenesis after acute ischemic stroke targeting VEZF1
Source: Aging (Albany NY). 2019 May 7;11(9):2762–86. doi: 10.18632/aging.101948 (PMC6535071; doi:10.18632/aging.101948)
Supplement: Supplementary Figures [file aging-11-101948-s001.pdf]

SUPPLEMENTARY FIGURES

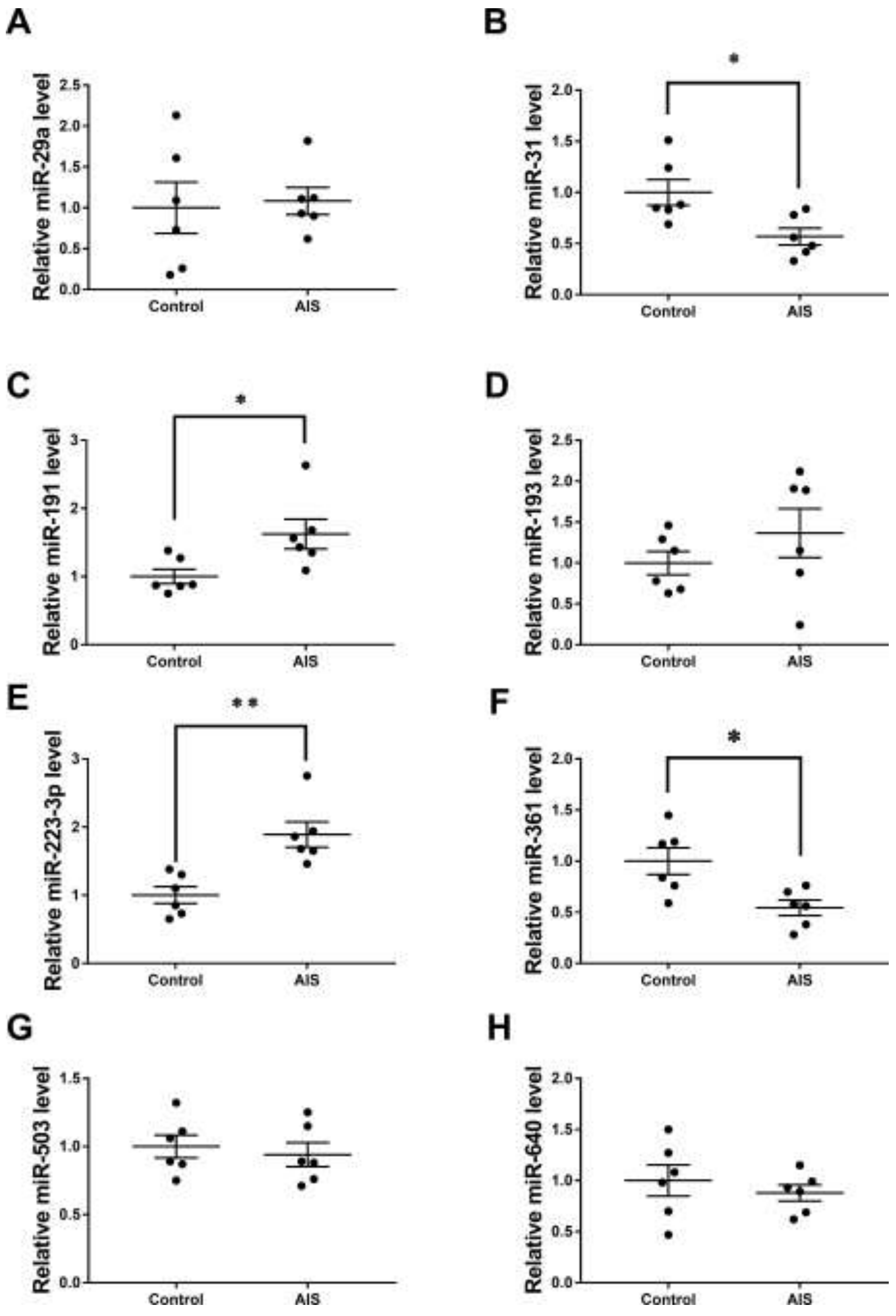

Supplementary Figure 1. Relative miRNAs levels. Expression levels of miRNAs in Cohort A (n=6) (**A**) miR-29a, (**B**) miR-31, (**C**) miR-191, (**D**) miR-193, (**E**) miR-223-3p, (**F**) miR-361, (**G**) miR-503, (**H**) miR-640.

南京医科大学附属逸夫医院　　　　　　　　　　　　伦理委员会

伦理审查批准件

项目伦审编号：2018-SR-25

试验项目名称	急性脑梗死患者外周血 miR-191 异常表达的作用及机制研究		
药物类别/期别	临床科研	申请专业	老年医学科
申办单位	南京医科大学附属逸夫医院	组长单位	
专业负责人	鲁翔	职务/职称	主任医师
主要研究者	鲁翔	职务/职称	主任医师
审查材料	试验方案，V1.0，版本日期：2018 年 12 月 10 日；知情同意书，版本号：02，版本日期：2018 年 12 月 28 日；研究者履历及参加人员列表	审查途径	■会议审查<br>□快速审查
本伦理委员会联系方式	地址：南京市龙眠大道 109 号南京医科大学附属逸夫医院<br>电话：025-87115593　　　邮箱：IRB@njmu.edu.cn		
伦理委员会列席人员签名	详见附表		
伦理委员会审评意见			
经本伦理委员会审查，同意进行该项临床试验。<br>意见及建议：■无□有<br>该研究的进行过程中将受伦理委员会的持续审查？■是□否<br>审查频度为研究批准之日起：□3 个月　□6 个月　■1 年<br>伦理审查委员会有权根据实际进展情况改变持续审查频度。<br><br>主任委员签名：[illegible]　　　日期：[illegible].01.02			

Supplementary Figure 2. Ethical approval of Ethics Committee of Sir Run Run Hospital, Nanjing Medical University (Protocol Numbers: 2018-SR-25)

南京医科大学 Institutional Animal Care and Use Committee (IACUC)

动物实验伦理审查同意书
Affidavit of Approval of Animal Ethical and Welfare

申请编号	11586	批准编号 Approval No.	IACUC-1806010

本《动物实验方案》经过实验动物伦理委员会审核，符合动物保护、动物福利和伦理原则，符合国家实验动物福利伦理的相关规定。方案的相关信息如下：

The animal use protocol listed below has been reviewed and approved by the Animal Ethical and Welfare Committee (AEWC).

实验名称 Protocol Title	大鼠大脑中动脉栓塞（MCAO）模型miRNA表达的研究<br>Expression of miRNAs in middle cerebral artery occlusion (MACO)				
申请人姓名 Applicant	杜康<br>Du Kang	职称/学位 Title/Degree	博士研究生<br>phD. student	邮箱 Email	dukang@njmu.edu.cn
实验负责人 Principle Investigator	鲁翔<br>Lu Xiang	职称/学位 Title/Degree	教授<br>professor	邮箱 Email	luxiang66@njmu.edu.cn
院系（部门） Department	附属逸夫医院老年医学科<br>Department of Geriatrics, Sir Run Run Hospital, Nanjing Medical University			申请日期 Application Date	2018-06-06
拟实验时间 Period of Protocol	2018-07-01 – 2020-06-01	实验动物使用许可证 Number of Animal Use Permit		SYXK（苏）2016-0016	
审核意见 Results of Inspection	☑符合动物福利伦理要求，可以进行实验。 Agree<br>☐调整方案后，可以进行实验。 Agree after modify				
兽医师 Chief Veterinary Officer	Zhangai hua			日期 Date	2018.6.15

南京医科大学实验动物福利伦理审查委员会
Animal Ethical and Welfare Committee of NJMU
主席(Chairman)：施爱民
日期(Date)：2018-06-15

地址：中国南京天元东路818号南京医科大学 邮编：211166
Add: Tianyun East Road 818, Nanjing Medical University, Nanjing, Jiangsu Province, P. R. China
1/11

Supplementary Figure 3. Ethical approval of Animal Ethical and Welfare Committee of Nanjing Medical University (Protocol Numbers: IACUC-1806010).

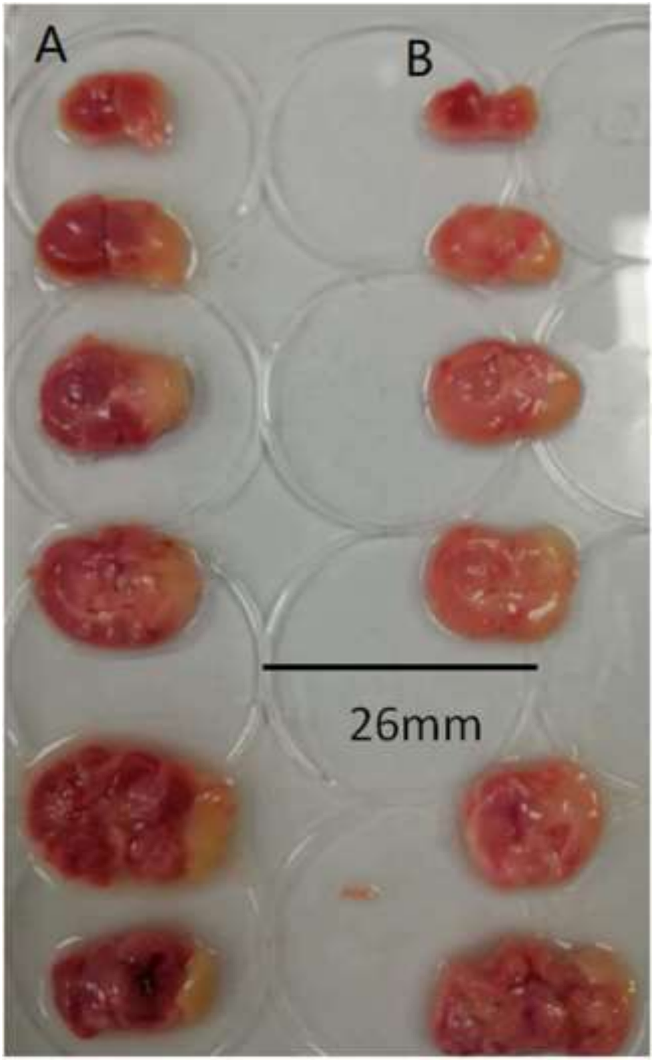

Supplementary Figure 4. TTC staining of the brains of rats MCAO.
